# *In Vivo* Protection against Strychnine Toxicity in Mice by the Glycine Receptor Agonist Ivermectin

**DOI:** 10.1155/2014/640790

**Published:** 2014-09-15

**Authors:** Ahmed Maher, Rasha Radwan, Hans-Georg Breitinger

**Affiliations:** Department of Biochemistry, The German University in Cairo, Main Entrance of Al Tagamoa Al Khames, New Cairo 11835, Egypt

## Abstract

The inhibitory glycine receptor, a ligand-gated ion channel that mediates fast synaptic inhibition in mammalian spinal cord and brainstem, is potently and selectively inhibited by the alkaloid strychnine. The anthelminthic and anticonvulsant ivermectin is a strychnine-independent agonist of spinal glycine receptors. Here we show that ivermectin is an effective antidote of strychnine toxicity *in vivo* and determine time course and extent of ivermectin protection. Mice received doses of 1 mg/kg and 5 mg/kg ivermectin orally or intraperitoneally, followed by an intraperitoneal strychnine challenge (2 mg/kg). Ivermectin, through both routes of application, protected mice against strychnine toxicity. Maximum protection was observed 14 hours after ivermectin administration. Combining intraperitoneal and oral dosage of ivermectin further improved protection, resulting in survival rates of up to 80% of animals and a significant delay of strychnine effects in up to 100% of tested animals. Strychnine action developed within minutes, much faster than ivermectin, which acted on a time scale of hours. The data agree with a two-compartment distribution of ivermectin, with fat deposits acting as storage compartment. The data demonstrate that toxic effects of strychnine in mice can be prevented if a basal level of glycinergic signalling is maintained through receptor activation by ivermectin.

## 1. Introduction 

The inhibitory glycine receptor (GlyR) is a glycine-gated chloride channel of the Cys-loop family of ion channel receptors. Currently, five GlyR subunits (α1–α4, *β*) are known, although only α1–α3 were found to be expressed in mammals [1–4]. Functional GlyRs consist of five subunits of homomeric (*α*_5_) or heteromeric (α/*β*) stoichiometry [2, 5]. GlyRs mediate rapid synaptic inhibition in spinal cord and brainstem and have been identified in higher brain areas, such as hippocampus, retina, or cochlea [1, 2]. Furthermore, glycine receptors are found presynaptically, where they are thought to modulate neurotransmitter release [1, 6].

The main agonist for the GlyR is the amino acid glycine [4, 7]; two other endogenous amino acids activate glycine receptors, namely, *β*-alanine and taurine. They bind to GlyRs to increase membrane chloride conductance [2, 7]. Glycine receptor-mediated currents are modulated by a variety of agents including alcohol, zinc, picrotoxin, and others [6, 8]. Strychnine, an alkaloid with a convulsive action extracted from the Indian tree* Strychnos nux vomica,* is a selective, highly potent (K_D_ 1–10 nM) antagonist of spinal glycine receptors [7]. It selectively blocks spinal postsynaptic inhibition [9, 10] through interaction with the N-terminal domain of the GlyR α subunit at a site distinct from but partially overlapping with the ligand-binding site [1, 5, 11, 12]. Competitive as well as a noncompetitive antagonism of GlyRs by strychnine has been reported [1, 5, 11–13].

Ivermectin (22,23-dihydroavermectin B1a) is a macrocyclic lactone that is widely used as an antiparasitic and anthelminthic drug in domestic animals [14–18]. In addition, it is considered the drug of choice for lymphatic filariasis and river blindness (onchocerciasis) in humans [19, 20]. Its antiparasitic action is mediated through the ivermectin-sensitive glutamate-gated Cl^−^ channel receptor α (DmGluClα) that is found in a number of invertebrate phyla [21]. Ivermectin is the only non-amino acid agonist of the inhibitory glycine receptor in mammals, activating the receptor ion channel by a glycine- and strychnine-independent pathway [22–24]. Moreover, ivermectin has an anticonvulsant action in a variety of vertebrate seizure models, thought to be mediated by GABA_A_ receptors [21]. Ivermectin was shown to be an effective antidote against lidocaine- and strychnine-induced convulsions. Non-lethal doses of strychnine in rats were effectively antagonised by ivermectin, with an ED_50_ of 4.25 mg/kg [25].

Here we show that ivermectin is able to protect mice against lethal doses of strychnine* in vivo* and describe the optimization of concentration, time course, and mode of application to achieve maximum protection against strychnine toxicity. Our data demonstrate that basal activation of glycine receptors through the independent agonist ivermectin is sufficient to neutralize the lethal effects of the specific receptor blockage by strychnine.

## 2. Materials and Methods 

### 2.1. Chemicals

Ivermectin (IVM, Sigma-Aldrich, Deisenhofen, Germany) was dissolved in DMSO to give a stock concentration of 100 mg/mL and was stored at −20°C. Directly before use, the stock was diluted into DMSO to the desired amount. The injected volume of DMSO (control group A) or ivermectin solution (experimental groups) was 50 *μ*L. All animals received the same amount of DMSO, which did not produce any notable effect. The small injection volume of 50 *μ*L was measured with an automatic pipette and delivered using an ultrafine insulin syringe. Strychnine (Sigma-Aldrich, Deisenhofen, Germany) was dissolved in working buffer (pH 7.4) at a stock concentration of 10 mM and stored in aliquots at −20°C. For experiments, the stock was diluted with working buffer to the desired concentration. Working buffer (concentrations in mM): KCl (5.3), NaCl (145), MgCl_2_ × 6H_2_O (1.7), CaCl_2_ × 2H_2_O (1.8), HEPES (25).

### 2.2. Animals

Male Swiss-Webster mice from El-Skary Animal Farm (Cairo, Egypt) weighing 25–30 g were housed in standard cages at 5 animals per cage at a temperature of 22 ± 2°C with a 12 h light/dark cycle. Animals had free access to food and water. All experiments were carried according to the guidelines of the Animal Care and Use of the Ethics Committee of the German University in Cairo. Animals were divided into different groups and were given strychnine and ivermectin according to the protocols described below. The number of animals per each group was kept at the minimum that allowed meaningful conclusions;* n*'s for each group are given in the figure legends.

### 2.3. Experimental Procedure

Animals were divided into one control group A and three major experimental groups. Control group A received an intraperitoneal dose of vehicle (50 *μ*L of DMSO). 30 min later, an ip injection of 2 mg/kg strychnine [26] was given and the time for tonic extensor convulsions (TEC) and death was recorded.

### 2.4. Systemic IVM

**Figure 1:**
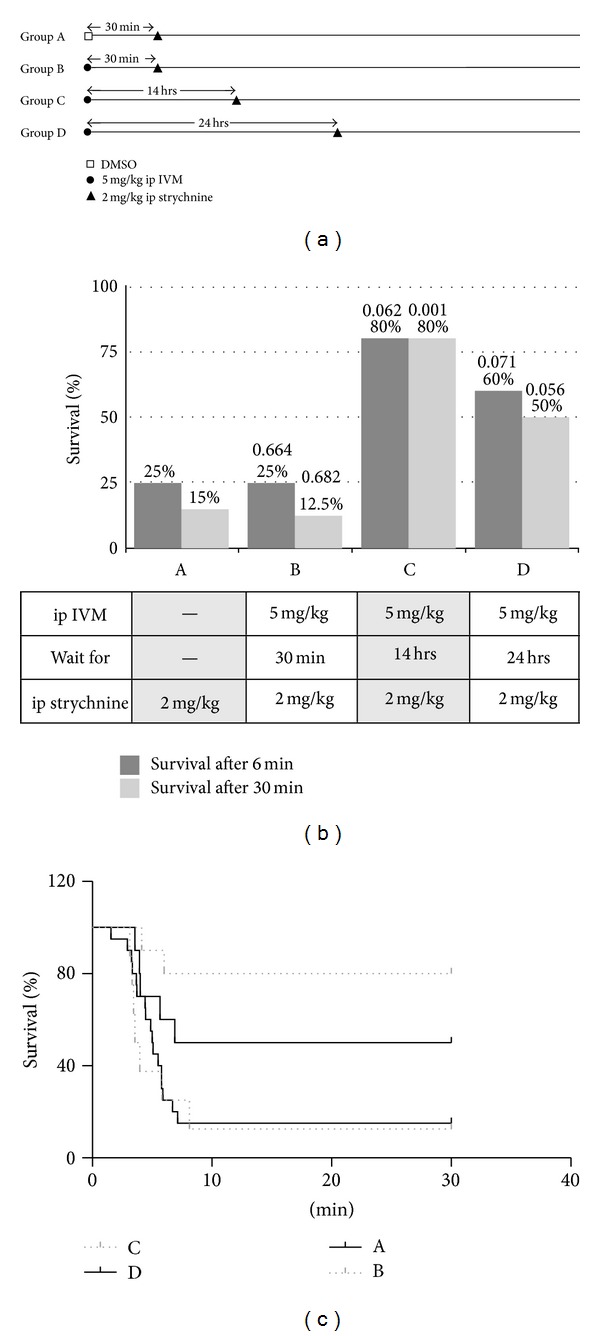
Protection against strychnine toxicity by intraperitoneal ivermectin. (a) Dosage schedule for systemic IVM administration. Group A received vehicle (DMSO, open square), groups B–D received 5 mg/kg IVM (solid circles), followed by 2 mg/k strychnine (sold triangles) after the indicated waiting period. Number of animals: A (*n* = 20); B (*n* = 8); C (*n* = 10); D (*n* = 10). (b) Survival rates of groups A–D. The *P* values for statistical significance (Fisher's one-tailed* t*-test), relative to control (group A) are given above each column. (c) Time course of survival in groups A–D.

To determine the time course of systemic IVM, mice were given 5 mg/kg ivermectin by ip injection. After varying time intervals, 2 mg/kg strychnine was given intraperitoneally and the time until TEC and death was recorded (Figure 1(a)).

### 2.5. Oral IVM

**Figure 2:**
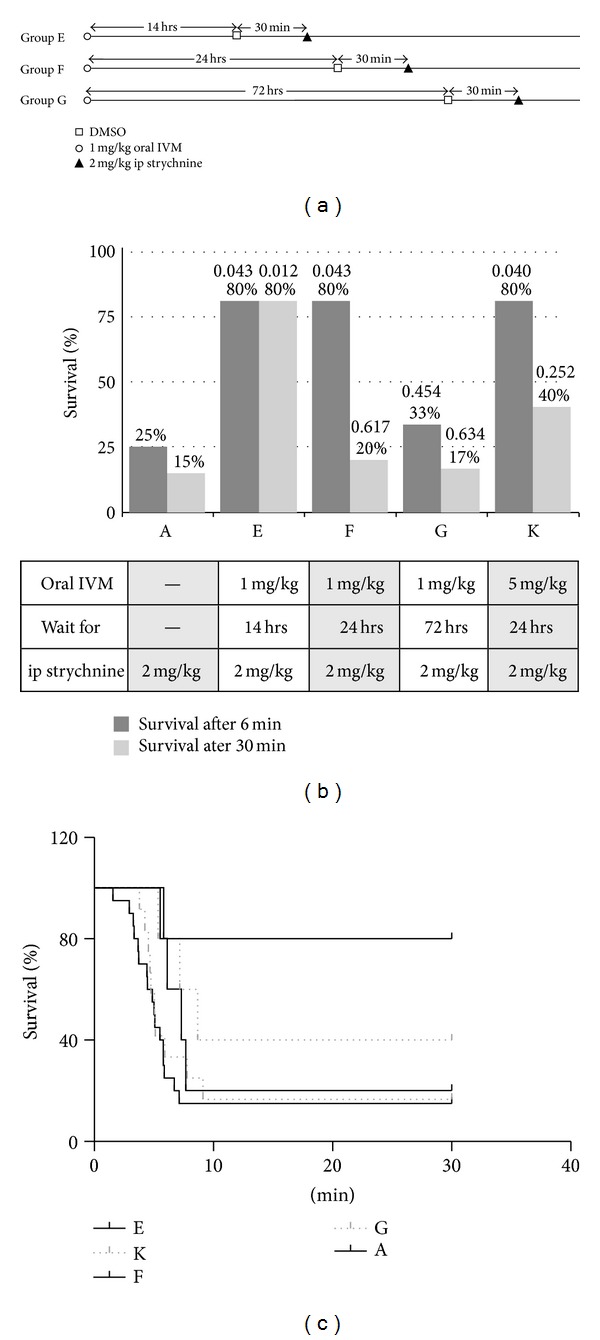
Protection against strychnine toxicity by oral ivermectin. (a) Dosage schedule for oral IVM administration. Groups E–G received 1 mg/kg IVM in DMSO (open circles), followed by vehicle injection (DMSO, open squares) after the indicated waiting time, and injection of 2 mg/k strychnine (sold triangles) after another 30 min. Number of animals: E (*n* = 5); F (*n* = 5); G (*n* = 12). (b) Survival rates of groups A–D. The *P* values for statistical significance (Fisher's one-tailed* t*-test), relative to control (group A), are given above each column. (c) Time course of survival in groups E–G. Survival curve for control group A (Figure 1(c)), receiving strychnine only, is added.

To test the effect of 1 mg/kg oral IVM, mice were given the oral dose followed by 2 mg/kg strychnine after a waiting time (varied). Time until TEC and death was recorded (Figure 2). A fourth group (K) was given 5 mg/kg rather than 1 mg/kg of ivermectin.

### 2.6. Combined Oral and Systemic IVM

**Figure 3:**
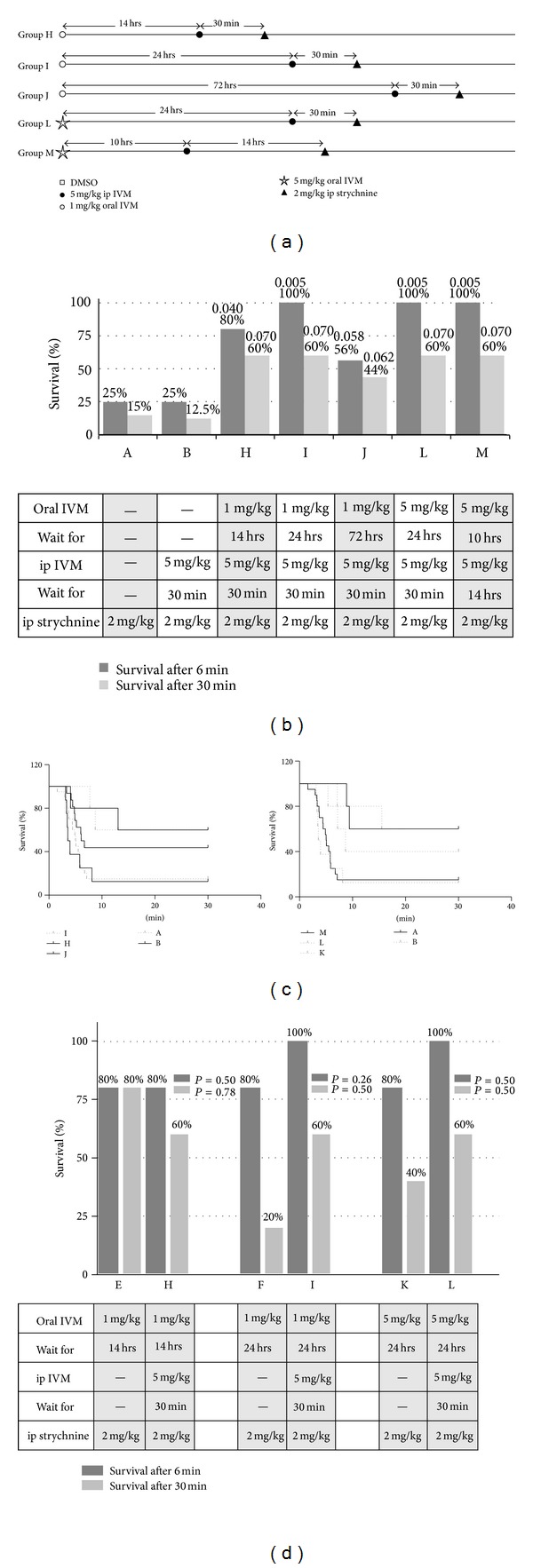
Combined effects of oral and systemic ivermectin administration. (a) Dosage schedule for combined IVM administration. All groups H–M received an oral ivermectin dose of 1 mg/kg (H–J, open circles) or 5 mg/k (L-M, open stars), followed by 5 mg/kg ip ivermectin (solid circles) after the indicated waiting times, and 2 mg/kg strychnine (solid triangles) after another 30 min (H–L) or 14 hrs (M). Number of animals: H (*n* = 5); I (*n* = 5); J (*n* = 16); K (*n* = 5); L (*n* = 5); M (*n* = 5). (b) Survival rates of groups H–M. For comparison, survival rates of control groups A (vehicle only) and B (5 mg/kg ivermectin only) are also shown. The *P* values for statistical significance (Fisher's one-tailed* t*-test), relative to control (group A), are given above each column. (c) Time course of survival in groups H–J (left panel) and K–M (right panel). Survival curves for control groups A and B are added for comparison. (d) Direct comparison of protective effects of a second dose of ivermectin after waiting times of 14 and 24 hours.

The combined effects of 1 mg/kg oral and 5 mg/kg ip doses of ivermectin were studied after different time intervals (Figure 3). Five groups of animals were tested, two of which received a higher oral dose of ivermectin (5 mg/kg rather than 1 mg/kg).

### 2.7. Statistics

Statistical significance for total number of protected animals of each group was determined using one-tailed Fisher's exact test. Survival curves were analyzed by the Mantle-Cox log rank test for significance and trend using Graphpad Prism version 5a (GraphPad Software, Inc., USA). A* P* value of 0.05 was considered significant in both tests.

## 3. Results

Doses of strychnine at 2 mg/kg were lethal in 85% of all mice tested (Figure 1). As tonic extensor convulsions were always followed by death, only time until death (*t*_*d*_) was used for analysis. In the control group A, *t*_*d*_ was always less than 6 min (one of 10 mice had *t*_*d*_ = 8 min), so 6 minutes was taken as a cut-off to determine whether IVM has a protective effect against strychnine. Prolongation of survival to >6 min was considered protective, as it clearly opposed the effects of strychnine. Mice that lived for more than 30 minutes were considered as surviving, as no strychnine toxicity was observed after this time. Notably, strychnine toxicity was rapid, with effects occurring within 10 minutes after application (mostly at <6 min). Protective effects of ivermectin on the other hand were only observed after waiting times of several (>10) hours.

### 3.1. Systemic IVM

**Figure 4:**
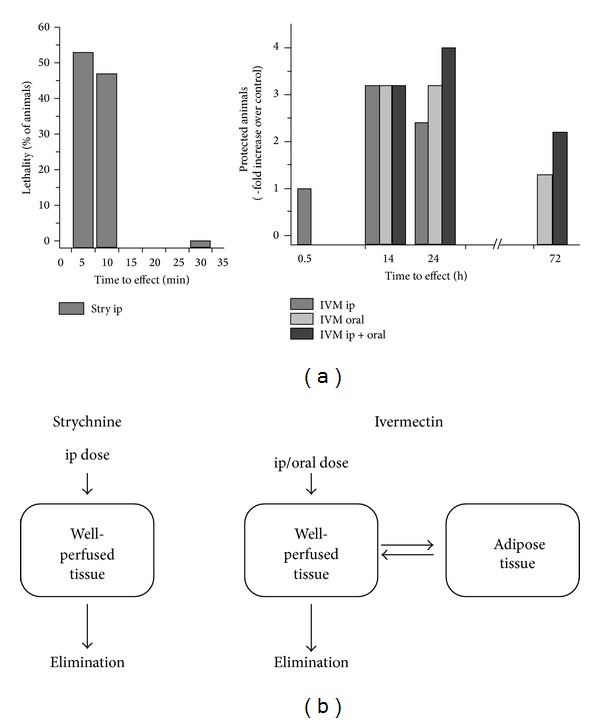
Time course of strychnine and ivermectin effects. (a) Time course of strychnine and ivermectin action. For strychnine, lethality (% of animals) within 0–5 and 5–10 min is plotted. After 30 min, no effects of strychnine were observed. For ivermectin, the -fold increase in survival time >6 min was plotted relative to control animals which received only vehicle. (b) One- and two-compartment models of distribution of strychnine and ivermectin in tissue.

Intraperitoneal ivermectin increased survival rate and survival time above the cut-off only after several hours of waiting (Figure 1, subgroups C and D). Intraperitoneal ivermectin given 30 min prior to strychnine challenge had no protective effect (Figure 1(b)). Increase in survival rates was maximal after 14 hours (80%) and still pronounced after 24 hours (50%). Notably, the protective effect of ip ivermectin was in an all-or-none fashion, *t*_*d*_ was not affected, and animals surviving for >6 min were completely protected. The time course of protection is also evident from survival analysis (Figures 1(c) and 4(a)). Protection against strychnine toxicity by ip ivermectin was significant according to the Mantle-Cox test (*P* = 0.004). The decline of protection after 24 hours is likely due to clearance of ivermectin from the cerebrospinal fluid.

### 3.2. Oral IVM

Application of ivermectin by the oral route had a protective effect after 14 hours (group E) and 24 hours, with survival prolonged to more than 6 minutes and an increased percentage of survivors (Figure 2). Maximum protection was observed after 14 hours (Figure 2(b), group E), and again survival was all-or-none. After 24 hours, protective effects declined, falling to control levels within ~72 hours (Figure 2(b), groups F and G). Survival analysis (Mantle-Cox test) showed that protection by oral ivermectin gave a strong trend (*P* = 0.056,* P* (trend) = 0.006). Likely, the reduced protection after 72 hours is due to clearance of the drug. Considering only the time points until 24 hours, where drug clearance was not notable, protection by oral ivermectin was statistically significant (*P* = 0.025). Increasing the oral dose of IVM from 1 mg/kg to 5 mg/kg (Figures 2(b) and 2(c), group K) gave a minor increase of protection against strychnine toxicity. Long-term survival (>30 min) increased from 20% to 40%; 80% of animals had survival time *t*_*d*_ of >6 min (Figure 2(b)). The increase in protection from the higher dose of ivermectin was not significant (*P* = 0.1048, Mantle-Cox-test) but showed a strong trend (*P* = 0.041). The time course of protection by ivermectin was similar for ip and oral route of administration with protective effects peaking at ~14 hours after ivermectin administration (Figure 4(b)).

### 3.3. Combined Oral and Systemic IVM

The effect of combining the oral and ip dose was studied in subgroups H, I, J, L, and M (Figure 3). After initial administration of ivermectin per os, and a waiting period of 14–72 hours, a second dose of ivermectin was given intraperitoneally, followed by the strychnine challenge 30 min later. In all groups, protective effects of the combined ivermectin treatment on *t*_*d*_ and long-term survival were observed. Survival time (Figures 3(b) and 3(c)) was prolonged compared to both control groups (group A, no ivermectin pretreatment; group B, only 1 mg/kg ivermectin ip 30 min before strychnine challenge) with *t*_*d*_ increasing from ~6 min to >10 min in 100% of tested animals (Figure 3(c)). Long-time survival rates also increased relative to control, reaching up to 60% (Figure 3(b)). Increasing the dose of oral ivermectin from 1 mg/kg to 5 mg/kg did not have any additional effect over the 1 mg/kg dose (Figure 3, subgroups L and M). Comparing the effects of a single oral dose of ivermectin with those of a combined oral and ip administration, a clear improvement by the second “booster” dose could be seen. For 1 mg/kg oral ivermectin, improvement of survival by a second dose of ip ivermectin was significant (subgroups F–I, *P* = 0.031, Mantle-Cox test) and also for an initial dose of 5 mg/kg ivermectin (subgroups K–L, *P* = 0.026). Although the waiting time after the second, ip, dose of ivermectin was short, *t*_*d*_ and long-term survival were increased for animals receiving dual doses of the drug. Improvement after waiting periods of 10 and 14 hours was smaller in comparison (groups E–H). The peak level of protection that was reached after 14 hours was not further improved by additional doses of ivermectin (Figure 4(a)).

## 4. Discussion

The inhibitory glycine receptor (GlyR) is one of the major mediators of rapid synaptic inhibition in mammalian spinal cord [1, 2, 5, 6]. It is the pharmacological target of the alkaloid strychnine, a potent (K_D_ 1–10 nM) and selective inhibitor [1, 2, 5, 6]. Indeed, strychnine toxicity is mediated exclusively through GlyRs, death being caused by arrest of breathing musculature. The widely used anthelminthic and anticonvulsant ivermectin [15, 16, 27–29] was shown to be an agonist of the GlyR, activating the receptor through a strychnine-independent pathway [22, 23]. Ivermectin was found to antagonise convulsions caused by nonlethal doses of lidocaine and strychnine in rats, suggesting that both GABAergic and glycinergic transmission were protected by ivermectin [25]. Here, we demonstrated and characterised the ability of ivermectin to protect mice from the toxicity of a lethal dose of strychnine, likely by providing sufficient basal activity of spinal glycine receptors to alleviate strychnine block. Ivermectin was administered at doses that were safe for the animals, but gave a clear biological effect. Doses of 1.3 mg/kg of ivermectin by oral administration are considered safe, while higher doses carry an increased risk of teratogenicity [30]. Intraperitoneal ivermectin had been used for mice at doses between 1.25 and 10 mg/kg [31], with a reported LD50 of 18 mg/kg [25]. The doses for ivermectin of 1 mg/kg (oral) and 5 mg/kg (ip) that we used were within the safe limits reported in the literature [25, 28–31].

When ivermectin was administered to mice prior to a lethal strychnine challenge of 2 mg/kg, protection against strychnine toxicity was observed. Protection was seen as (i) prolonged survival (>6 min) after strychnine administration and (ii) increase in the percentage of mice who survived the potentially lethal dose of strychnine altogether, that is, survival longer than 30 min, after which time the toxic effects of strychnine were no longer observed (Figure 4).

The kinetics of distribution within the body are different for strychnine and ivermectin. Strychnine inhibition of GlyRs was fully developed after 5-6 minutes. After 30 minutes, the toxic effects of strychnine were no longer observed (Figure 4(a)). In contrast, ivermectin distributes much more slowly to the glycine receptor sites. When given 30 min after ip administration, 1 mg/kg ivermectin showed no protective effect (Figure 1(b)), while after 14 hours the same dose caused a survival rate of 80%. After 24 hours the protective effect of a single ip dose was lower than at 14 hours (Figure 1(b)). The rapid time course of strychnine toxicity would agree with a single compartment distribution of the substance [32–34]. Ivermectin is known for its slow kinetics of distribution and excretion [16]; measurable amounts of the drug in humans and domestic animals are still found up to 17 days after administration of a single dose [16, 35, 36]. The ivermectin molecule is highly hydrophobic and is considered to accumulate extensively in fatty tissue which acts as a reservoir (Figure 4(b)), causing slow clearance from the body [16]. Thus, ivermectin distribution can be described by a two-compartment model [34], which is compatible with the time course we observed for ivermectin action. Kinetics of ivermectin administered per os were similar to those observed with intraperitoneal administration (Figures 2 and 4) with maximum protection after 14 hours of administration and consecutive loss of activity after this time. This finding is in agreement with the pharmacokinetics of ivermectin observed in goats, where administration per os or subcutaneously resulted in moderate differences in plasma concentration, but similar antiparasitic efficacy [36].

Increasing the dose of ivermectin from 1 mg/kg to 5 mg/kg produced a moderate increase in protection. Combining ip and oral routes of administration, a further increase in survival (improved protection against strychnine toxicity) was observed, particular at times >24 hours, that is, after the peak of activity, when excreted drug was replenished by a second dose. Our observation that kinetics of ivermectin protection were similar for oral and intraperitoneal routes of administration (Figure 4) agrees with a two-compartment model for the distribution of ivermectin.

Overall, our data show that ivermectin can protect mice against strychnine toxicity* in vivo*. Strychnine selectively and exclusively targets spinal glycine receptors, disrupting their synaptic signaling. Protection by ivermectin likely results from the basal, strychnine-independent activation of glycine receptors. This “nonsynaptic” basal activation appears to produce enough glycinergic signal to prevent the lethal myoclonic attacks observed in strychnine poisoning. Indeed, restoration of some inhibitory synaptic activity has been also shown to be effective in the glycine receptor-associated disorder hyperekplexia (OMIM 149400), which can be treated with GABAA receptor agonists [7]. The strychnine-resistant basal activity of glycine receptors mediated by ivermectin did not produce any notable side effects in the animals and yet was sufficient to protect against strychnine toxicity. The combination of slow pharmacokinetics and a broad therapeutic window is useful for systemically acting drugs.* In vivo* protection of GlyRs may be therapeutically relevant in the treatment of glycine receptor-associated motor disorders, such as hyperekplexia, stiff man syndrome, and other convulsant diseases.
